# Differences between laboratory scanner and intra-oral scanner regarding axes and distances of three implants in a curved line when using two types of intra-oral scan bodies: in vitro study

**DOI:** 10.1186/s40729-025-00617-7

**Published:** 2025-04-01

**Authors:** Gil Ben-Izhack, Diva Lugassy, Joseph Nissan, Fatmi Safadi, Tal Shirazi, Yifat Manor, Asaf Shely

**Affiliations:** 1 Dental Division, Shamir (Assaf Harofeh) Medical Center, 70300 Zerifin, Israel; 2 Department of Orthodontics, The Maurice and Gabriela Goldschleger School of Dental Medicine, Faculty of Medical and Health Sciences, Tel Aviv University, 6997801 Tel Aviv, Israel; 3 Department of Oral Rehabilitation, The Maurice and Gabriela Goldschleger School of Dental Medicine, Faculty of Medical and Health Sciences, Tel Aviv University, 6997801 Tel Aviv, Israel; 4 Private Laboratory, Habarzel 44, Tel Aviv, Israel; 5 Department of Oral and Maxillofacial Surgery, The Maurice and Gabriela Goldschleger School of Dental Medicine Faculty of Medical and Health Sciences, Tel Aviv University, 6997801 Tel Aviv, Israel

**Keywords:** Digital dentistry, Implant axis, Dental implants, Intra-oral scan body, Laboratory scanner, Intra-oral scanner, Oral rehabilitation

## Abstract

**Background:**

The objective of this study was to evaluate differences in the intra-implant distance, inter-implant distance, intra-implant axis and inter-implant axis of two different intra-oral scan bodies (ISBs) which are connected to three implants in a curved line by comparing laboratory scanner (LBS) versus an intra-oral scanner (IOS).

**Methods:**

Printed model with three internal hexagon implant analogs at the locations of 12#, 13#, and 14# was produced. Two ISBs, MIS Dentsply Sirona (MIS) and Zirkonzhan (ZZ), with different geometries (MIS trapezoid, ZZ cylindrical) were scanned one time by using LBS (master model) followed by thirty scans with IOS. After each scan a stereolithography (STL) file was produced and each IOS STL file was superimposed with the LBS STL file (master model) by using three-dimensional (3D) analysis software PolyWorks^®^2020. A Kolmogorov–Smirnov test was performed followed by a Mann–Whitney test (*p* < 0.05).

**Results:**

Mean errors for inter-implant distance were significantly lower for MIS compared to the ZZ (*p* < 0.05). In contrast, mean errors for intra-implant angle were significantly lower for ZZ compared to MIS (*p* < 0.05). Mean error for inter-implant angle was significantly lower for MIS compared to ZZ only between 12# to 14# and no difference was found between the other couples (*p* < 0.05).

**Conclusions:**

ISB geometry influenced all four parameters: intra-implant distance, intra-implant angle, inter-implant distance and inter-implant angle. MIS ISB trapezoid geometry resulted significantly lower mean error regarding most parameters except intra-implant angle. ZZ ISB cylindrical geometry had a good impact only on the intra-implant angle.

**Supplementary Information:**

The online version contains supplementary material available at 10.1186/s40729-025-00617-7.

## Introduction

The digital workflow provides us with a comfort technique for reducing chair time, improving patient comfort, reducing impressions and plasters distortions (especially for mobile teeth) and creating digital files which can be restored for many years [1–4]. Digital impressions can be produced by direct or indirect techniques. The direct technique is by using an intra-oral scanner (IOS) in the clinic and sending a digital file to the lab, while the indirect technique is by using conventional impression which is sent to the lab where the technician scans it by using a laboratory scanner (LBS) [5]. When scanning implants located in partial edentulous arches by using the direct technique with Primescan (CEREC^®^ Primescan; Dentsply Sirona, Milford, DE, USA), whether anterior 4-unit region or posterior 3-unit region, both accuracy and time efficiency is acceptable [6, 7]. As technology advances the accuracy of the direct technique, when using IOS, it also provides an acceptable and accurate method as same as the conventional impressions method for achieving a solid result when scanning implants in a partially edentulous region [8]. However, when examining partially edentulous regions, differences can be found between straight and curved lines due to several factors such as: scanning technique, types of intra-oral scan bodies (ISBs), types of IOS, type of different software's and user experience [8–11].

Stereolithography (STL) file is produced after scanning with either IOS or LBS, the three-dimensional (3D) main objective is to precisely capture the geometry of an object. STL file is a computer aided design (CAD) mesh, its purpose is to transmit a 3D structure with the highest degree of accuracy [12].

A global standard for accuracy is described at ISO 5725, as accuracy is composed of two measuring methods (ISO, 5725-2: 2019): (1) Trueness, which refers to how close the measurements are regarding to a reference/master model or to the actual dimension of an object which is measured; (2) Precision, which is the consistency of the scans or the closeness between the obtained results of a tested object [13, 14].

LBS is still considered as gold standard compared to IOS due to its higher accuracy [15, 16].

Capturing the 3D position of an implant requires an ISB which can be composed of different materials or combinations of them. One of the most common materials are polyether ether ketone (PEEK) and titanium type 5, the use of a metallic ISB is preferred over polymer due to less wear after multiple use and sterilization process [17, 18].

ISB geometry is differ between the commercial companies, it can be cylinder, conical or trapezoid, it can also be from one-piece or two-piece and all these characteristics may influence the accuracy when trying to capture implant position [19–22].

The position of the ISB regarding the dental arch may also influence the accuracy, when scanning a full arch compared to a partially edentulous space the accuracy is deteriorating and it is interesting to examine whether a curved line may affect this issue [21, 22].

Capturing the true position of an implants is important for achieving the best passive fit for the restoration. When multiple implants are used it is important to capture the relative position between the implants as most accurately as possible and it is known from the literature that the curve at the anterior region of the jaw may influence and cause scanning errors [23–25], these errors regarding distances and angles between ISBs influence directly on passive fit, hence, it has an important clinical consequence in everyday dentistry when using IOS for capturing implant 3D location while using ISBs.

In the early 90’s Jemt [26] suggested that an adequate passive fit will produce a restoration with no clinical problems for the long term. Since there is a difference between tooth movement (50–200 μm) and an implant movement (3–50 μm) Brunski suggested that the upper limit for passive fit restoration should be 100 μm and a lack of passive fit will lead to strains and micromovement which may damage the bone around the implant [27, 28].

It is difficult quantifying passive fit of an implant framework, while Brånemark [29] stated that misfit for implant framework should not exceed 10 μm, Klineberg and Murray [30] proposed that a gap of 30 μm is acceptable as it does not exceed 10% of the implant circumference, one true value does not exist and a range of 10 µm to 150 µm misfit is considered clinically accepted in the literature [31, 32].

The objective of this study was to evaluate differences in the 3D position (intra-implant distance, inter-implant distance, intra-implant angle, and inter-implant angle) of two ISBs with different geometries, which are connected to three implants in a curved line (locations #12, #13, #14) by superimpose STL files (LBS versus IOS). The null hypothesis was that no difference will be found between the two ISBs regarding all parameters (intra-implant distance, inter-implant distance, intra-implant angle, and inter-implant angle).

## Materials and methods

**Fig. 1:**
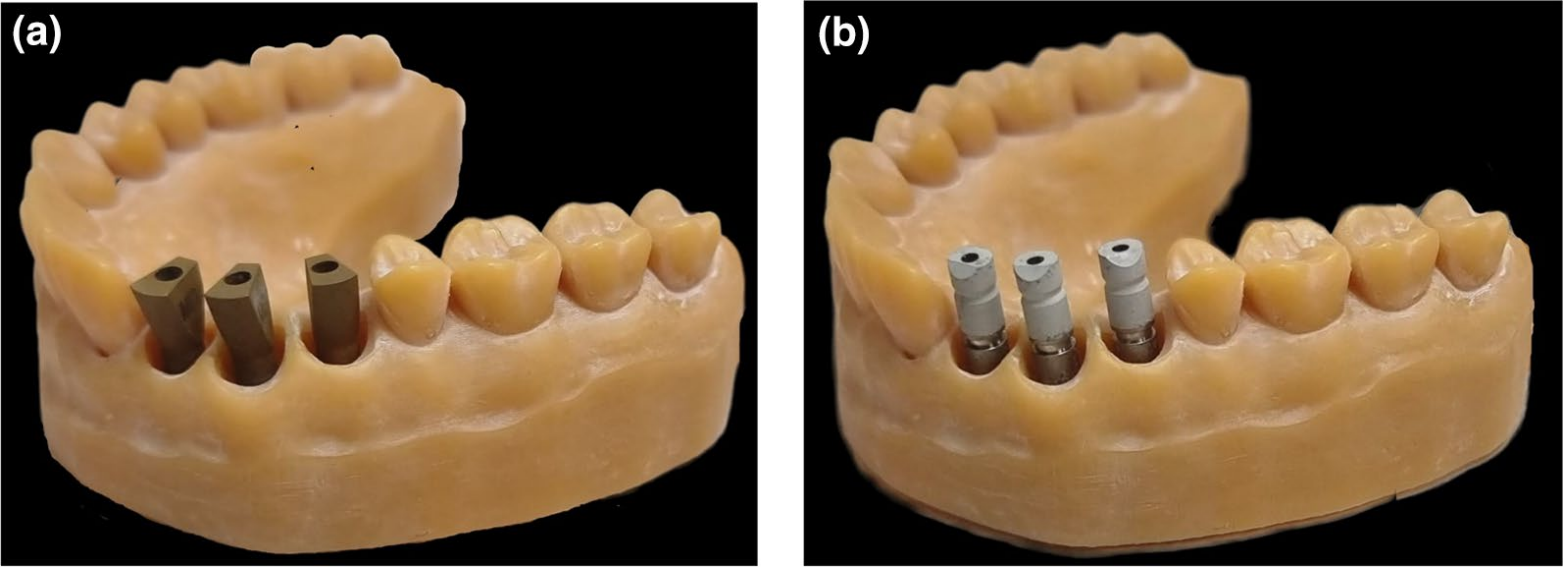
**a** MIS ISBs inserted in place of teeth 12, 13, 14. **b** ZZ ISBs inserted in place of teeth 12, 13, 14

For a laboratory in-vitro design a 3D resin model was created by using a SolFlex 650X50 printer (VOCO GmbH, Heidelberg, Germany). MIS standard internal hexagon implant analogs, length 11.5 mm and diameter 3.75 mm, were inserted in the locations of teeth #12 (1),#13 (2),#14 (3), as in the clinic this is a valid treatment option for missing teeth 12,13 and 14. Two different ISBs were used (Fig. 1):

1. MIS ISB (MIS, Titanium, two piece), internal hexagonal connection, trapezoid /asymmetrical geometry.
2. Zirkonzahn ISB (ZZ, Titanium, two piece), internal hexagonal connection, cylindrical/asymmetric geometry.

The insertion of both MIS ISB and ZZ ISB into the analogs was by using electronic implant prosthetic screwdriver iSD900 (NSK^®^, Osaka, Japan) at 15 N.cm. A master model was created for both ISBs as the models were scanned using 7 Series dental wings (Dental wings^®^, Montreal, QC, Canada) desktop laboratory scanner (LBS). A QR file was produced and converted to STL file. The laboratory scanner is defined as gold standard regarding accuracy and only one scan is enough since this is defined as master model [16]. Primescan (CEREC^®^ Primescan; Dentsply Sirona, Milford, DE, USA) IOS was used for creating the intra-oral scans, scanning protocol was done as suggested by Dentsply Sirona. For each ISB thirty scans were done and thirty STL files were exported (CEREC^®^ Connect Software 5.2.6; Dentsply Sirona, Milford, DE, USA), one for each scan. By using digital software (PolyWorks^®^2020; InnovMetric, Québec QC, Canada) with the best-fit method a super imposition of each IOS scan to the LBS scan (master model) was performed (30 times for each ISB).

**Fig. 2:**
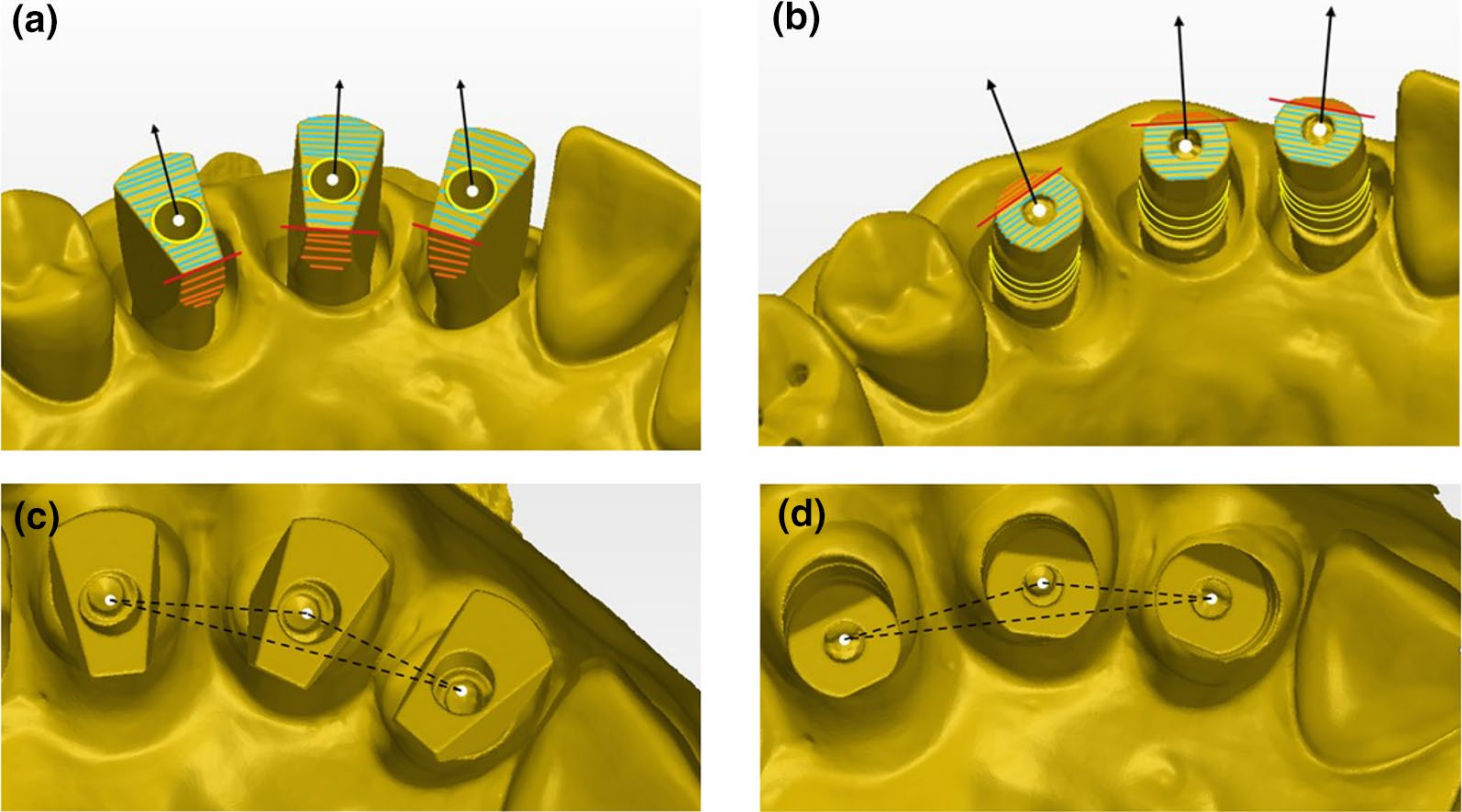
**a** MIS cylinder, upper plane, central point, axis, side plane, and sideline. **b** ZZ cylinder, upper plane, central point, axis, side plane, and sideline. **c** MIS inter-implant distance. **d** ZZ inter-implant distance

The geometry of the two ISBs is different, yet both have a flat surface, MIS ISB is pointed to the lingual surface and ZZ ISB is pointed to the buccal surface (Fig. 1). The three ISBs were numbered as the mesial was defined as number 1 (#12), the middle as number 2 (#13), and the distal as number 3 (#14). The special geometry of both ISBs is an advantage as it is easier for overlapping the STL files. By using PolyWorks Inspector™ 2020 Software Verification and Measurement (PolyWorks^®^2020; InnovMetric, Québec QC, Canada) a superimposition (best-fit algorithm) of the LBS STL (master model) with each one of the IOS STL (thirty times for each ISB) was performed. Before the super imposition process and for analyzing all measurements, many definitions were applied. As a result, calculations of the spatial characterization of each ISB relative to the master model (both distances and axes) were done. The following definitions are (Fig. 2):

- Cylinder (yellow line)—Occlusal cylinder of MIS ISB and outer gingival cylinder of ZZ ISB.
- Upper plane (green plane)—Occlusal surface of the ISB.
- Central point (white dot)— Point of intersection between the cylinder and the upper plane of the ISB.
- Axis (black arrow)—Longitudinal axis of the associated best-fit cylinder to the ISB.
- Side plane (orange plane)—Associated best-fit side plane of the ISB.
- Side line (red line)—Intersection between the upper plane and the side plane, also used for defining the system of axes of each ISB.
- Inter-implant distance (black intermitted line)—Distance between two central points: distance 1–2, distance 2–3, and distance 1–3. The difference between master model and each scan was calculated by subtraction.

**Fig. 3:**
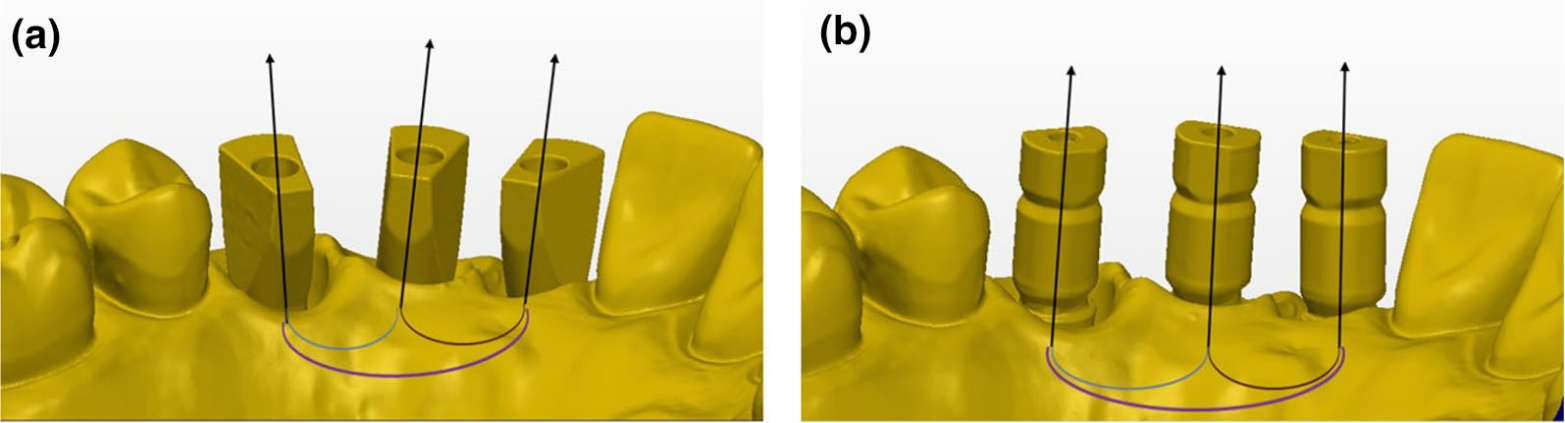
**a** MIS intra-implant and inter-implant angles. **b** ZZ intra-implant and inter-implant angles

Delta axis 1–2, 2–3, and 1–3 was defined as the angle formed between the axes of each two ISBs: (Figure 3)

h. Delta axis 1–2 (blue)—Angle formed between the axis of the mesial and middle ISBs.
i. Delta axis 2–3 (brown)—Angle formed between the axis of the middle and distal ISBs.
j. Delta axis 1–3 (purple)—Angle formed between the axis of the mesial and distal ISBs.

The difference between master model and each scan was calculated by subtraction.

**Fig. 4:**
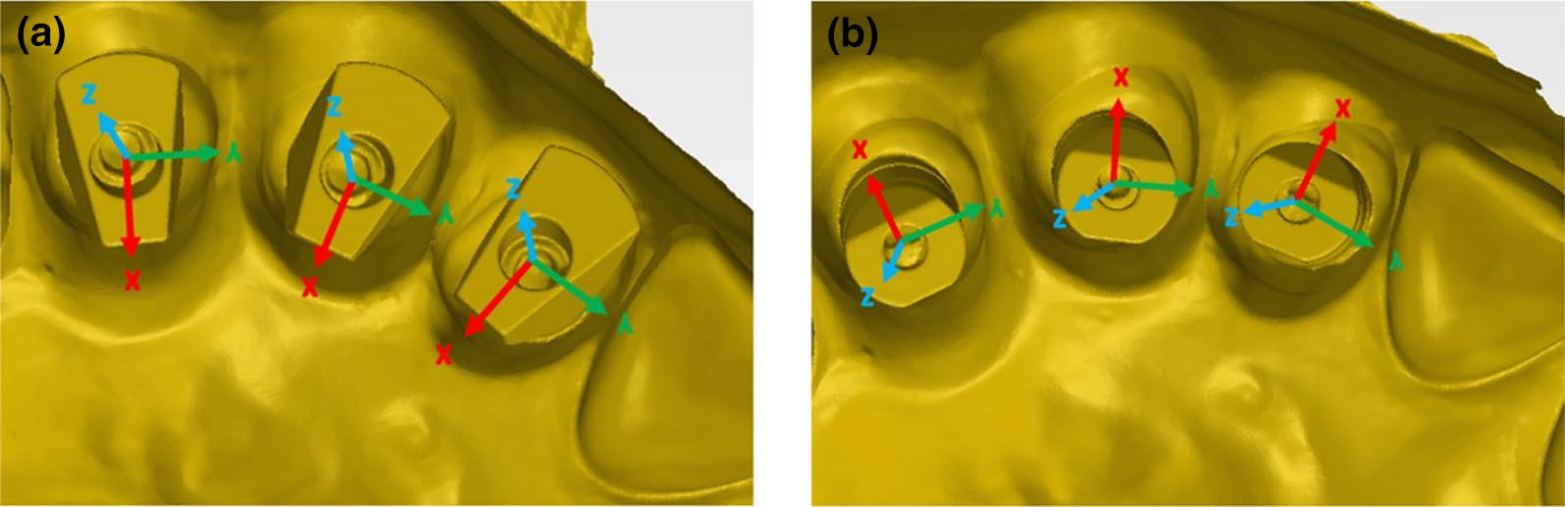
**a** MIS x, y, z axis system. **b** ZZ x, y, z axis system

In each master model STL axis system was defined and the center was located at the central point (Fig. 4):

1. X-axis (red)—Buccal–lingual, positive direction is towards buccal.
2. Y-axis (green)—Mesial–distal, positive direction is towards distal.
3. Z-axis (blue)—Occlusal–gingival, positive direction is towards occlusal.

After superimposition of the LBS STL file and IOS STL file (thirty times for each ISB) measurements of axes and distances were done. Calculations of the spatial characterization of both MIS ISBs and ZZ ISBs relative to the master model were as follows:

The distance between each central point of the LBS scan and its parallel central point of the IOS scan was defined as the shift in the ISB head (both MIS and ZZ) between the LBS scan and the IOS scan from all the axes (white dot). This was calculated as follows:

1. D central point _1,2,3_ = $$\sqrt{{X}^{2}+{Y}^{2}+{Z}^{2}}$$
2. Delta axis _1,2,3_—The 3D angle difference between each longitudinal axis (Axis) of the LBS scan and its parallel longitudinal axis (Axis) of the IOS scan.

The following measurements were calculated and used for statistical analysis:

1. Intra-implant distance (mm)—Changes between the D central points in each single ISB.
2. Inter-implant distance (mm)—Changes between the D central points of each two ISBs.
3. Intra-implant angle (angle)—Changes between the delta-axis in each single ISB.
4. Inter-implant angle (angle)—Changes between the delta-axis of each two ISBs.

A statistical analysis was conducted by using Statistical Package for Social Sciences for Windows Release 23.0 (SPSS Inc., Chicago, IL, USA). By applying a Kolmogorov–Smirnov test on the study variables no normal distribution was received. A Mann Whitney test with 30 samples in each group would be sensitive to the effect of Cohen’s d = 0.753 with 80% power (alpha = 0.05, two tailed). due to this result Mann–Whitney tests were used for examining differences between MIS ISB and ZZ ISB groups. The statistical significance level for this work was *p* < 0.05.

## Results

A Kolmogorov–Smirnov test was performed on the study variables indicating no normal distribution (*p* < 0.05).

Table 1 presents the mean error (Mean), standard deviation (SD), range (Range), and percentiles (P25, P50, P75) for both ISBs (MIS and ZZ) regarding four parameters: intra-implant distance (1, 2, 3), inter-implant distance (12, 23, 13), intra-implant axis (1, 2, 3), and inter-implant axis (12, 23, 13).

**Table 1:** Mean error; ± SD; range; P25 (25th percentile), P50 (50th percentile), and P75 (75th percentile) regarding Intra-implant distance (1, 2, 3), Inter-implant distance (12, 23, 13), Intra-implant angle (1, 2, 3) and Inter-implant angle (12, 23, 13) for both MIS and ZZ ISBs

	MIS ISB				ZZ ISB			
	Mean (± SD) Range	P25	P50	P75	Mean (± SD) Range	P25	P50	P75
Intra-implant distance 1 (mm)	0.039 (± 0.033) 0.153	0.019	0.027	0.045	0.049 (± 0.071) 0.255	0.014	0.018	0.051
Intra-implant distance 2 (mm)	0.035 (± 0.031) 0.116	0.016	0.026	0.045	0.068 (± 0.113) 0.542	0.018	0.027	0.041
Intra-implant distance 3 (mm)	0.034 (± 0.042) 0.232	0.016	0.022	0.030	0.062 (± 0.066) 0.258	0.032	0.037	0.049
Inter-implant distance 12 (mm)	− 0.009 (± 0.028) 0.157	− 0.018	− 0.004	0.006	0.029 (± 0.126) 0.731	0.000	0.009	0.014
Inter-implant distance 23 (mm)	− 0.000 (± 0.115) 0.052	− 0.009	0.002	0.009	0.059 (± 0.241) 1.370	0.007	0.0145	0.020
Inter-implant distance 13 (mm)	− 0.008 (± 0.027) 0.143	− 0.023	− 0.004	0.008	0.018 (± 0.022) 0.134	0.011	0.018	0.029
Intra-implant angle 1 (angle)	0.667 (± 0.714) 3.667	0.287	0.446	0.815	0.353 (± 0.333) 1.445	0.143	0.233	0.469
Intra-implant angle 2 (angle)	0.593 (± 0.570) 3.129	0.291	0.439	0.722	0.377 (± 0.586) 2.306	0.064	0.165	0.303
Intra-implant angle 3 (angle)	0.478 (± 0.294) 1.753	0.320	0.462	0.578	0.468 (± 0.579) 2.393	0.159	0.238	0.525
Inter-implant angle 12 (angle)	0.050 (± 0.580) 3.425	− 0.291	0.044	0.339	− 0.125 (± 0.255) 1.417	− 0.239	− 0.165	-0.076
Inter-implant angle 23 (angle)	− 0.071 (± 0.307) 1.142	− 0.257	0.004	0.186	0.022 (± 0.140) 0.606	− 0.104	− 0.013	-0.088
Inter-implant angle 13 (angle)	0.043 (± 0.640) 2.812	− 0.143	0.159	0.389	− 0.135 (± 0.464) 2.539	− 0.289	− 0.232	-0.155

Mann–Whitney tests examined the differences between MIS ISBs and ZZ ISBs by comparing the mean errors between the IOS scans to the LBS scan (master model).

**Fig. 5:**
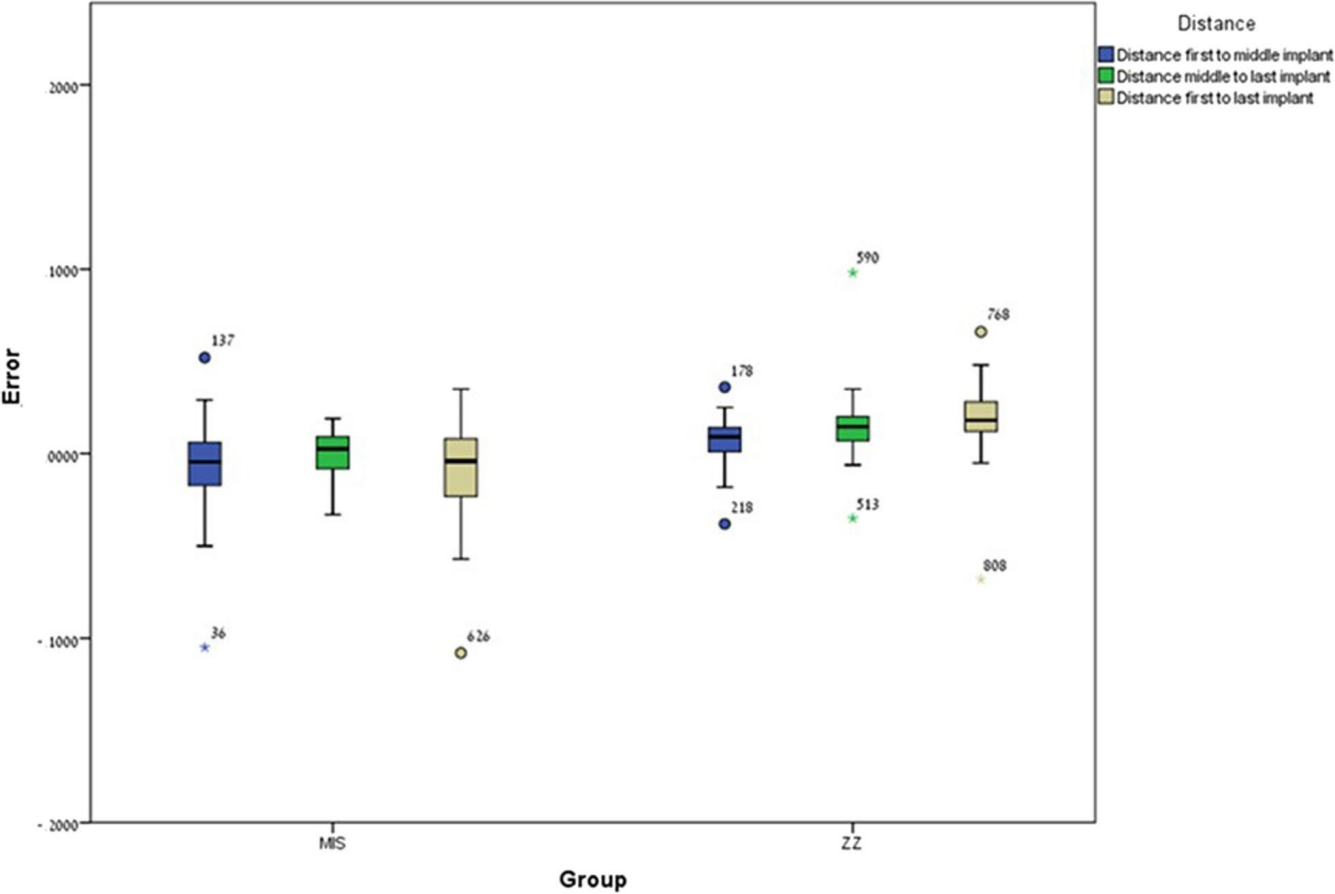
Inter-implant distance mean error and ± SD for ZZ ISB and MIS ISB

Significant differences were found between ZZ ISB and MIS ISB regarding inter-implant distance 12 (*p* = 0.001), inter-implant distance 23 (*p* = 0.0005) and inter-implant distance 13 (*p* = 0.0005). For all variables the mean error for MIS ISB was lower compared to ZZ ISB (Fig. 5).

**Fig. 6:**
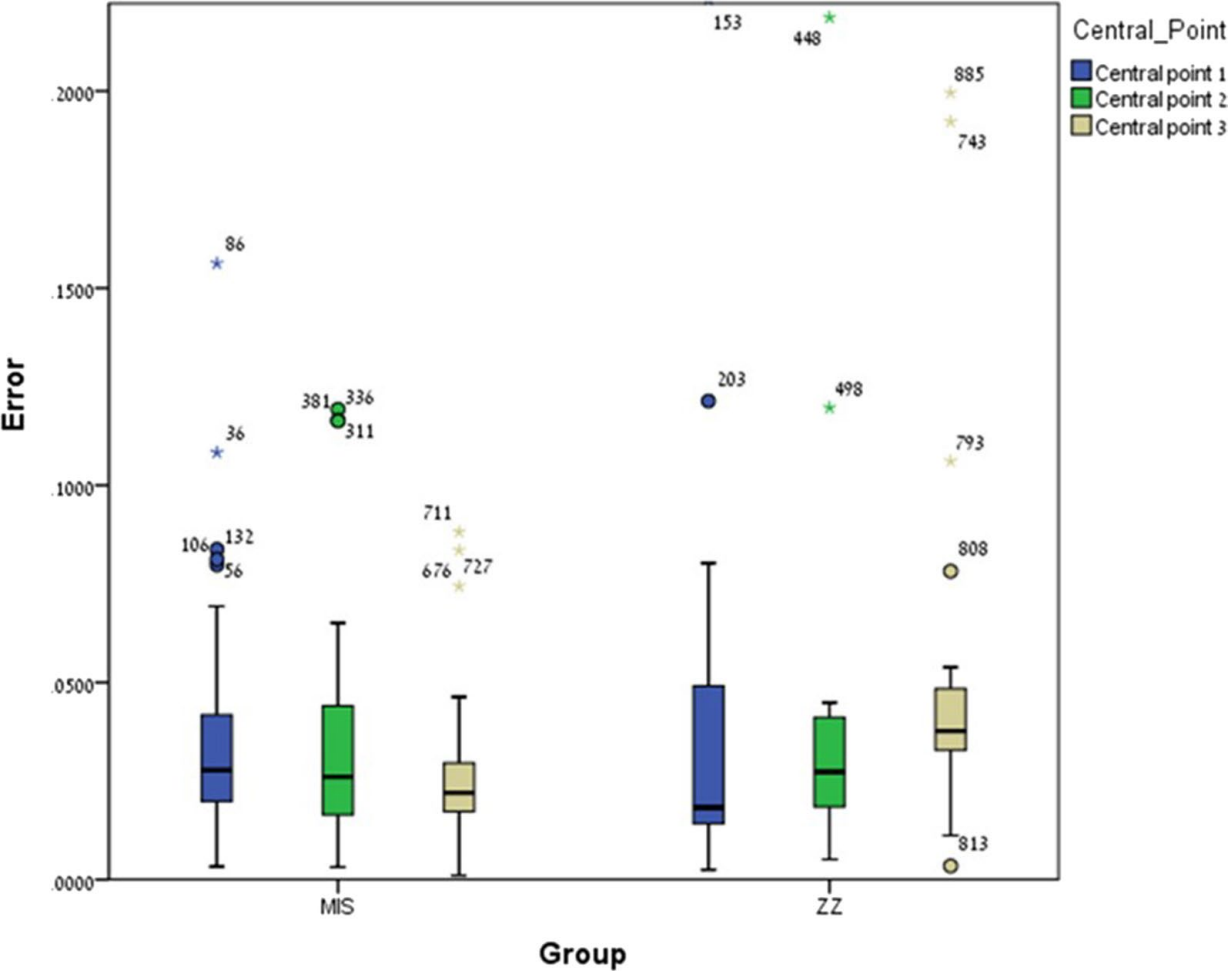
Intra-implant distance (central points 1, 2, 3) mean error and ± SD for ZZ ISB and MIS ISB

No significant differences were found between ZZ ISB and MIS ISB regarding central point 1 (*p* = 0.186) and central point 2 (*p* = 0.416) with exception to central point 3 (*p* = 0.001) in which the mean error among MIS ISB was significantly lower compared to ZZ ISB (Fig. 6).

**Fig. 7:**
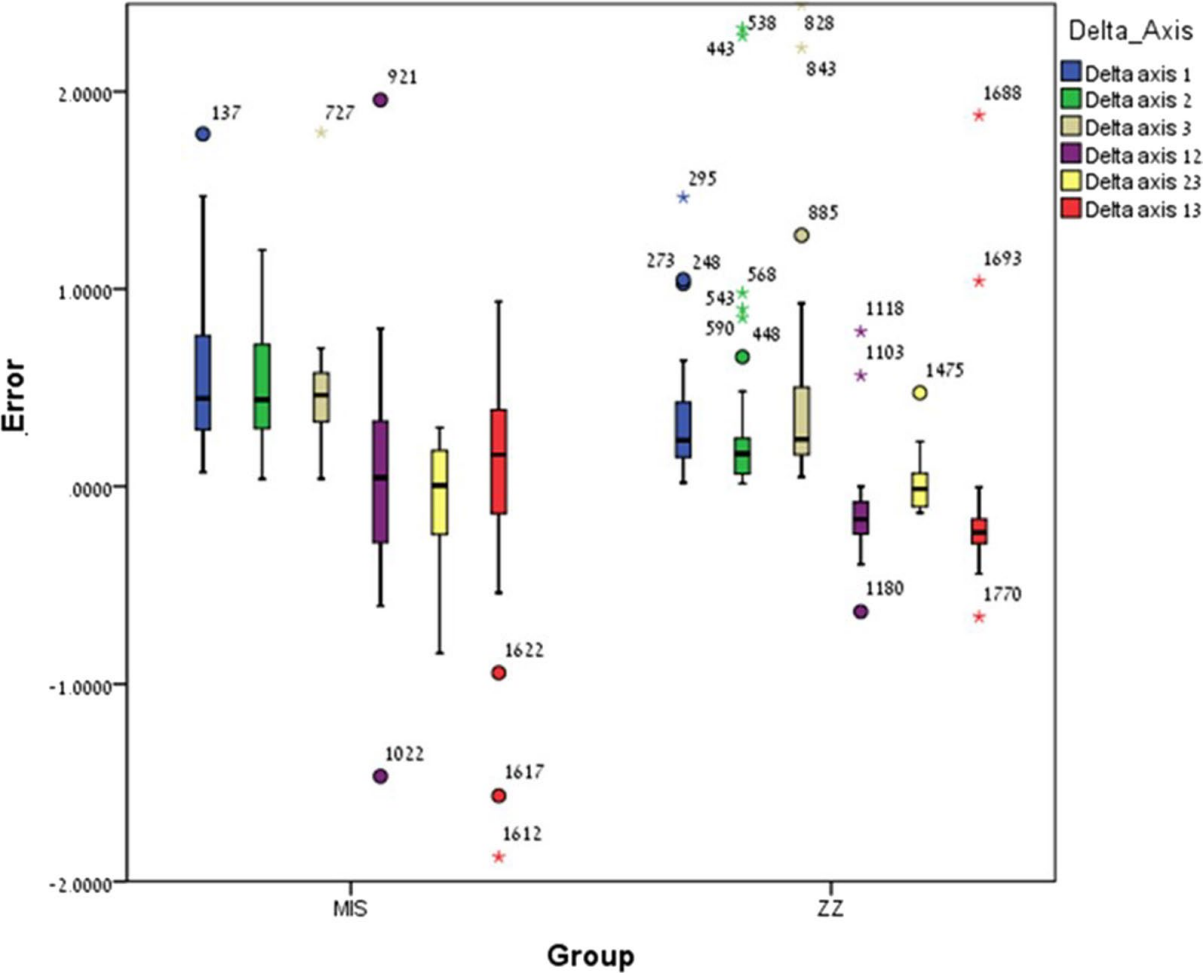
Intra-implant angle and inter-implant angle (delta axis 1, 2, 3 and delta-axis 12, 23, 13) mean error and ± SD for ZZ ISB and MIS ISB

Significant differences were found between ZZ ISB and MIS ISB regarding Delta axis 1 (p = 0.009), Delta axis 2 (p = 0.002) and Delta axis 3 (p = 0.018) as the mean error of ZZ ISB was lower compared to MIS ISB. However, no significant differences were found for Delta axis 12 (p = 0.098) and Delta axis 23 (p = 0.853) with exception of Delta axis 13 (p = 0.001) in which the mean error among MIS ISB was significantly lower compared to ZZ ISB (Fig. 7).

## Discussion

In this study a previous method [9, 17] was used for measuring differences (mean errors) in several parameters between two different ISBs when comparing LBS scan (master model) to IOS scan by superimposition of STL files.

The tested ISBs showed significantly different mean errors regarding all four examined parameters (inter-implant distance, intra-implant distance, inter-implant angle, and intra-implant angle), hence, our null hypothesis was rejected.

When examining the inter-implant distance, the MIS ISB had significantly lower mean errors compared to the ZZ ISB. Although both ISBs are two-piece titanium their shape is different as the MIS ISB is trapezoid, has sharp angles and a wider occlusal surface area compared to the ZZ ISB which is cylindrical and rounded. Several studies which investigated the connection between inter-implant distance and scanning errors concluded that as the distance between the ISBs increases the mean error increases as well (accuracy decreases) [33–35]. These results agree with the results of this current study as the MIS ISB which has a larger upper surface compared to the ZZ ISB hence reduces the distance between the ISBs and provides lower mean errors regarding inter-implant distance. It is also important to mention that due to the trapezoid geometry of the MIS ISB it is possible to reduce the inter-implant distance by screwing it with different hex positions, while it is not possible when using ZZ ISB which has a cylindrical shape.

The intra-implant angle exhibited significantly lower mean errors for the ZZ ISB compared to the MIS ISB. Since the ZZ ISB has cylindrical shape, it can be assumed that it was easier for the best-fit algorithm to superimpose the STL files regarding to the longitudinal axis itself. This result is consistent with our previous study where a straight line was examined with the same ZZ ISB [36].

For the inter-implant angle the MIS ISB had significantly lower mean error compared to the ZZ ISB only regarding delta axis 13, no difference was found for delta axis 12 and delta axis 23. It is interesting that for the longest distance MIS ISB had significantly lower mean errors compared to ZZ ISB. This again can be assumed regarding the geometry of the ISB. The results of the mean inter-implant angle for both ISBs are within the reported results in the literature (0.07–0.3°) [37].

The intra-implant angle is an important factor also for passive fit, Andriessen et al. reported that a deviation of more than 0.194 degrees for an implant length of 14.8 mm is beyond the acceptable limit as it may create 50 μm difference at the apex of the implant, it was also mentioned that between two implants the threshold of inter-implant distance should be below 100 μm and deviation of less than 0.4 degrees [38]. In this study, for inter-implant distance the mean errors were all below 100 μm and inter-implant angles were all below 0.4 degrees, therefore, curved line fix-partial dentures fabricated with the tested scans from this study would end up in a clinically acceptable fit.

These results have important clinical implications because it is known from the literature that inaccuracies, when capturing the 3D position of implants may create a prosthesis which has inaccurate occlusal and interproximal contact points, misfit of the restoration and these problems will increase the need for chairside or laboratory adjustments and cause functional or esthetic problems. If passive fit and occlusal contacts are not appropriate it may cause various complications in the long-term including abutment screw loosening, fracture of the restoration and implant fracture [39, 40].

IOS geometry, whether one-piece or two-piece, is a very investigated theme in literature and it is already known that geometry has an impact on the accuracy of intra-oral scans [41–43]. A study by Pan et al. [42] showed that the accuracy of digital scanning depends on the geometry and that noises and disruptions may be caused by sharp edges. In this study MIS ISB has sharp edges compared to the ZZ ISB but most of the results, especially regarding inter-implant distance and inter-implant angle, were favorable for the MIS ISB.

For this study two ISBs were used, both are made from two-piece titanium, there is still controversy in the literature regarding the effect of the material on the accuracy as some studies report that PEEK is less accurate compared to titanium [43, 44] while others report the opposite [45, 46].

When using different software programs for 3D analysis it may also lead to different results. Son et al examined differences between four 3D analysis software programs (Geomagic control X, GOM Inspect, Materialise 3-matic and Cloudcompare) when scanning full arch, half arch and single tooth preparation. Reference model was scanned one time with IOS (Solutionix C500; MEDIT, Seoul, Korea) and 20 scans were made for comparison with other IOS (CS3600; Carestream, Atlanta, GA, USA). Significant differences between the programs were found when scanning single tooth preparation and half arch, but when scanning full arch, no significant differences between the four programs were found. In the limitations, the author suggests that when using 3D software, it is essential to use the default values which are recommended by the manufacturer of each software program otherwise the values which are received may vary greatly [47]. For this study, only one program was used, full arch was scanned as suggested by Son et al. and the default values for best fit algorithm were used carefully as recommended by the manufacturer.

A method which has previously been used, was selected in this study for measuring mean errors regarding four parameters (inter-implant distance, intra-implant distance, inter-implant angle, and intra-implant angle) for comparing differences between two ISBs located in a curved line, comparison was made by superimpose STL files between LBS scan (master model) and IOS scans [9, 17].

Limitations of this in vitro study must be mentioned as the scans were in controlled conditions [48], but patients related factors were absent. The 30 scans made with the IOS for both ISBs were taken consecutively, and this may influence the accuracy of the IOS, this is a point which needs more research in the literature. Resin models were used and not metallic [49]. Only one IOS and LBS were used, and only two types of ISBs. The model in this study had three parallel implants [50]. The software used for superimposing the STL files in this study (PolyWorks^®^2020; InnovMetric, Québec QC, Canada, and best-fit method) has been previously reported [9, 17, 36]. Further in vitro and in vivo studies regarding the fit of fixed partial dentures on implants in a curved line are needed for corroborating the findings of this in vitro study when examining partially edentulous regions.

## Conclusions

1. ISB geometry influenced all four parameters: intra-implant distance, intra-implant angle, inter-implant distance and inter-implant angle.
2. MIS ISB trapezoid geometry showed significantly lower mean error regarding most parameters except intra-implant angle.
3. ZZ ISB cylindrical geometry showed significantly lower mean error regarding intra-implant angle.
4. Further research examining the influence of ISB geometry on accuracy is needed.

## Supplementary Information

Additional file 1.

Additional file 2.

## Data Availability

The datasets used and/or analyzed during the current study are available from the corresponding author on reasonable request.

## Abbreviations

ISBs: Intra-oral scan bodies
LBS: Laboratory scanner
IOS: Intra-oral scanner
STL: Stereolithography
3D: Three-dimensional
PEEK: Polyether ether ketone
MIS: MIS Dentsply sirona
ZZ: Zirkonzhan
CAD: Computer aided design
